# Regional Variation of Hospitalization Rates for Asthma in Korea: Association with Ambient Carbon Monoxide and Health Care Supply

**DOI:** 10.3390/ijerph17041244

**Published:** 2020-02-14

**Authors:** Agnus M. Kim, Sungchan Kang, Jong Heon Park, Yoon Kim

**Affiliations:** 1 Department of Health Policy and Management, Seoul National University College of Medicine, Seoul 03080, Korea; agnus@snu.ac.kr; 2 Graduate School of Public Health, Seoul National University, Seoul 08826, Korea; rjmcmc@gmail.com; 3 National Health Insurance Service, Wonju 26464, Korea; parkjh@nhis.or.kr; 4 Institute of Health Policy and Management, Medical Research Center, Seoul National University, Seoul 03080, Korea

**Keywords:** asthma, hospitalization, pollutants, carbon monoxide, bed, income, Korea

## Abstract

This study was performed to investigate the relationship between the hospitalization rate for asthma and the ambient carbon monoxide (CO) by examining regional variation of the hospitalization rates for asthma in Korea and its factors. The hospital inpatient claims for asthma were acquired from the National Health Insurance database in 2015. A multivariate linear regression was performed with the hospitalization rate for asthma as a dependent variable. The annual ambient concentration of CO showed a negative association with the hospitalization rates for asthma while that of sulfur dioxide showed a positive association. The number of primary care physicians showed a negative association with the hospitalization rates for asthma while the number of beds in hospitals with less than 300 beds showed a positive association. The negative association of the ambient concentration of CO with the hospitalization rates for asthma showed results upon further investigation.

## 1. Introduction

Asthma is the most common chronic respiratory disease, affecting 5% of the population globally [1]. As asthma is best understood as an inflammatory disease where immunologic reactions are involved, environmental factors, which are known to induce immunological reactions, have received increasing attention [2]. Concerning this, air pollutants are tempting subjects to study given their well-established association with respiratory diseases [3] and the increase in asthma prevalence in the latter half of the 20th century [4], which seems in-pace with an increase in air pollutants. However, despite accumulating evidence that suggests a positive association between air pollutants and asthma incidence, their association remains indefinite in terms of consistency among study results and specificity of their relationship [4,5,6].

Among the pollutants, the relationship between carbon monoxide (CO) and asthma requires further investigation. While a number of studies have documented a significant association between CO density and elevated mortality and hospital admission [7,8,9], the association with respiratory diseases is ambiguous with mixed findings [10,11]. The study of the relationship between CO and asthma is more limited. While the adverse impact of CO on asthma was observed in some studies [12,13,14], it was reported that an association between the ambient CO density and asthma was unlikely [15,16]. However, given the growing body of research favoring the therapeutic effect of CO on respiratory tract diseases in the population [11,17,18] and in preclinical conditions [19,20,21], the relationship between CO and asthma needs to be further investigated.

Asthma is the third leading cause of disease burden in Korea [22] with its increasing and relatively high prevalence compared with other developed countries [23,24,25]. Although the impact of air pollution on population health in Korea has been studied in asthma [26,27], as well as in other diseases [28,29], the influence of CO density remains to be investigated. This study first described the regional variation of the hospitalization rates for asthma in 251 districts divided into three age groups. Second, with the hospitalization rate for asthma in each district as a dependent variable, this study examined how the ambient CO was related to asthma when health-care supply factors, which were known to be highly related to hospitalization rates due to other common conditions [30,31], were adjusted. Meanwhile, this study investigated what might have resulted in the large difference in hospitalization rates for asthma in Korea.

## 2. Materials and Methods

This study was granted a waiver of informed consent and of its documentation by the Institutional Review Board of Seoul National University Hospital (IRB No: 1504–075-665). The hospital inpatient claims were acquired from the National Health Insurance (NHI) database in 2015. The NHI database covers the claims of the entire population of Korea. As used in the NHI claims in 2015, the Korean Standard Classification of Diseases, Sixth Revision codes (J 45: Asthma, J46: Status Asthmaticus) were used to identify the hospitalizations for asthma. The hospitalization rates for asthma were calculated per 10,000 population according to the 252 districts. Hospitalization rates were age- and sex-standardized to the Korean resident population of 2015 and calculated for three age groups additionally (aged 0–14, 15–64, and 65 and older).

The total population of Korea in 2015 was 51,014,947 with an average age of 40.2. The population size of the district, a basic administrative unit in Korea, ranged from 10,524 to 668,415 with an average of 204,733 in 2015. The average male to female ratio of the districts is 100.6, the same as that of the entire Korean population.

The variables used for the analysis are as follows: First, for a socio-demographic variable, the average NHI premium was used. The NHI premium is determined in proportion to the income of each household and is considered a reliable proxy measure for income [32,33]. Second, given the characteristic of asthma as a disease usually treated at a primary care level, we used the number of primary care physicians per 10,000 population. As there is no institutionally defined role of primary care physician in Korea, we used an operational definition, which identified primary care physicians by the characteristics of patients who visited the clinics [34]. The primary care physicians were defined as physicians in the clinics where the proportion of the visits for 52 simple and minor disease groups [35] was above the average (38.3%) of total clinics [36]. Third, given the prevailing influence of bed supply on hospitalization rates, the number of hospital beds per 1000 population was included as an independent variable. In order to differentiate the influence of hospital beds according to the sizes of hospitals, the number of beds was classified according to the size of the hospital (the number of beds in hospitals with less than 300 beds for small to medium sized hospitals and the number of beds in hospitals with more than 300 beds for large sized hospitals). The data for health-care resources were acquired from the Health Care Resources & Service Information Report issued by the Ministry of Health and Welfare [37].

Lastly, concerning ambient air pollutants, besides the ambient concentrations of CO, sulfur dioxide (SO_2_) and particulate matter 10 (PM_10_) were included based on prior literature [4,38]. The levels of air pollutants were measured in air monitoring stations located at each district, which were operated by local governments [37]. After being examined by the government-run environmental office and institutes, they are recorded in the database of the National Institute of Environmental Research. We used the annual average of the values measured monthly. For missing values of the districts without air monitoring stations, the values were imputed by averaging those in neighboring areas [39].

We primarily performed a Pearson correlation analysis among the variables and performed a multivariate linear regression with the hospitalization rates for asthma as dependent variables. In order to accurately assess the relationship between the ambient CO and the hospitalization rates for asthma, other potential factors were included as explanatory variables. All analyses were conducted using SAS, version 9.3 (SAS Institute, Inc., Cary, NC, USA) and SPSS 23 (IBM Corporation, Armonk, NY, USA).

## 3. Results

**Figure 1:**
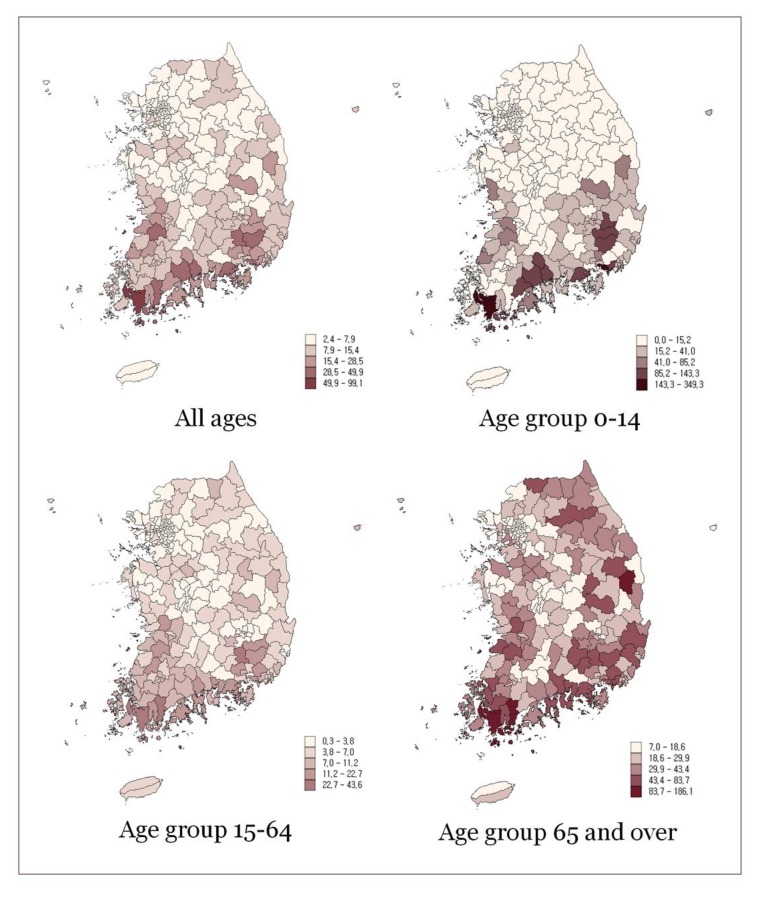
Hospitalization rates per 10,000 for asthma by age group.

There were a total of 40,319 hospitalizations due to asthma in Korea in 2015, which was 8.4 per 10,000 population. 33,098 hospitalizations were for Asthma and 9921 for Status Asthmaticus. The rate was the highest in the age group 65 and over at 24.7 per 10,000, and it was 15.6 and 4.2 in the age groups 0–14 and 15–64 respectively (Table 1). Though the total rate was higher in females than in males, in the age group under 14, males showed higher hospitalization rates. The regional distribution of hospitalization rates for asthma is presented in Figure 1 and Table 2. There was a 12 fold variation in the hospitalization for asthma, and the variation was most prominent in the age group 0–14 with a 22-fold difference among districts.

The correlation analysis is presented in Table 3. The average NHI premium showed weak negative correlations with the hospitalization rates for asthma, and the number of primary care physicians had weak negative correlations with the asthma hospitalization rates except for age group 0–14. The number of beds in small to medium sized hospitals showed a moderate positive correlation with the hospitalization for asthma. Among the air pollutants, only the ambient concentration of CO had a negative correlation with the hospitalization rates except for age group 0–14.

Table 4 shows the results of a multivariate linear regression analysis of the hospitalization rates for asthma. The characteristics of independent variables are in Table A1. While the concentrations of CO in all districts were below the pollution standard, those of SO_2_ exceeded the standard in more than half of the districts. In addition, the concentration of PM_10_ exceeded the standard in all districts [40]. The increase in the number of primary care physicians was associated with a decrease in hospitalization rates for asthma, and the increase in the number of beds in hospitals with less than 300 beds was related to an increase in hospitalization rates for asthma. The average NHI premium, unlike the correlation analysis where the hospitalizations rates for asthma showed a negative correlation in all age groups, showed a negative association only in the age groups 15–64 and 65 and over. The average annual ambient concentration of CO showed a negative association with the hospitalization rates for asthma, while that of SO_2_, showed a positive association.

## 4. Discussion

This study investigated the geographic distribution of the hospitalization rates for asthma in Korea and its association with the ambient CO and other factors. The number of primary care physicians showed a negative association with the hospitalization rates for asthma while the number of beds in hospitals with less than 300 beds showed a positive association. The annual ambient concentration of CO showed a negative association with the hospitalization rates for asthma while that of SO_2_ showed a positive association.

The hospitalization rates for asthma among age groups show a similar trend with the prevalence of asthma: highest among children, which is followed by the elderly. However, the regional difference in the hospitalization rates is far above the difference in the prevalence of asthma in Korea [25], and the variation in the hospitalization rates for asthma is unusually large compared with hospitalization rates of other common causes, which are also considered large compared to other countries [31,41]. This suggests that the decision for hospitalization for asthma in Korea is highly discretionary. In particular, a 22-fold difference in the age group 0–14 strongly suggests the likelihood that the hospitalization for asthma is determined by factors which are not related to the medical conditions of patients. The positive association between the hospital bed supply and the hospitalization rates found in this study supports this, which will be discussed further in the latter part of the discussion.

The association between poverty and hospitalization rates for asthma has been well investigated among children [42,43,44,45] and adults [44,46,47]. However, in our study, this relationship appeared in the age group 15–64 only slightly. Our results suggest that the socio-economic condition of a region has little effect on the hospitalization rates for asthma when the factors related to health service supply and ambient air pollutants are adjusted. Considering that no multicollinearity was detected among the variables, this diminishment of the effect of socio-economic deprivation of a region indicates that other factors have a stronger impact on the hospitalization rates for asthma.

The negative association between the hospitalization rates for asthma and the number of primary care physicians suggests the likelihood that the hospitalization for asthma declines if it is properly cared for at the primary care level. This relationship has been documented in studies about the hospitalizations for ambulatory care sensitive conditions which included asthma [31,48,49]. However, since those studies were not performed separately for asthma, our results can be considered to show more clearly the negative association between the PCP supply and the hospitalization rate for asthma. The positive association between the hospital bed supply and the hospitalization rates for asthma can be understood likewise. As in the prior studies about the ambulatory care sensitive conditions in Korea [30,31], the positive association was observed only with the hospital beds in the small to medium sized hospitals. This result, while reflecting the overcrowding and competitive nature of the current supply of beds in those hospitals, shows the existence of supply induced demand.

The ambient concentration of CO showed a negative association with the hospitalization rates for asthma, and this relationship occurred in all age groups, though statistically insignificant in the age group 0–14 (*p* = 0.93). The negative association between the ambient CO concentration and the hospitalization rate for asthma suggests the possibility that the CO can have an inhibitory effect on the occurrence or exacerbation of asthma, which is in line with prior studies suggesting the therapeutic effect of CO on respiratory diseases [17,18,19,20,21]. In particular, given the paucity of the finding of the protective effect of ambient CO on respiratory diseases [11], this study can be considered as evidence suggesting a protective role of CO on respiratory diseases as well as asthma. The fact that the CO concentrations in all districts were below the pollution standard [40] makes the protective effect of CO on inhibiting asthma found in this study more reliable.

In contrast with the case of CO, a positive association was found between the ambient SO_2_ and hospitalization rate for asthma. The impact of SO_2_ on asthma is ambiguous with some findings showing harmful effects [50,51] and others with no significant relationships [13,52,53,54]. This heterogeneity of finding may result from the diverse methods of analysis and study conditions. Despite that, the positive association found in this study deserves attention in that the SO_2_ concentration in Korea is high compared with other developed countries [55] and that, in many regions in Korea, the ambient SO_2_ concentration is above the standard for pollution [40]. The health effect of SO_2_ in asthma should be further investigated in future studies. Our study has several limitations. First, the unit of analysis of this study is a region. Therefore, the relationship between the independent variables, especially ambient air pollutant concentration and the hospitalization rates for asthma, may not necessarily mean the same relationship in individuals. Given this study design, the known individual factors, such as obesity and smoking, could not be included in the analysis. The fact that the concentration of ambient air pollutants was an averaged value lessens the accuracy of the study design. Most of all, this study was not a longitudinal study, which may be essential in investigating the causal relationship between air pollutant exposure and hospitalization for asthma. Therefore, our study results should be interpreted with caution and be followed by studies with a more sophisticated design. Despite these limitations, the negative associations between hospitalization rates for asthma and ambient CO concentration may serve as a precursor for further investigation of the inhibitory and therapeutic effect of CO on asthma. In addition, the possible overuse of hospitalization in asthma induced by supply of beds should be addressed.

## 5. Conclusions

The large variation in asthma hospitalization rates and a positive association between hospitalization rates for asthma and the number of hospital beds suggests the possibility of inducement of hospitalization for the disease, while the negative association of the number of primary care physicians and the hospitalization rates suggests the importance of primary care in managing the disease. Most of all, negative association of the ambient concentration of CO with the hospitalization rates for asthma should result in further study of the therapeutic effect of CO.

## Figures and Tables

**Table 1:** Hospitalization rates per 10,000 for asthma by age group and sex.

	Rate–Total (N. of Hospitalizations)	Rate–Male (N. of Hospitalizations)	Rate–Female (N. of Hospitalizations)
Hospitalization rate due to asthma per 10,000 (all ages)	8.4 (43,019)	6.8 (17,199)	10.1 (25,820)
Hospitalization rate due to asthma per 10,000 (0–14)	15.6 (11,173)	17.6 (6,491)	13.5 (4,682)
Hospitalization rate due to asthma per 10,000 (15–64)	4.2 (15,671)	2.9 (5,443)	5.6 (10,228)
Hospitalization rate due to asthma per 10,000 (65 and over)	24.7 (16,175)	19.2 (5,265)	28.7 (10,910)

**Table 2:** Variation statistics of hospitalization rates for asthma by age group.

	Mean	Max	Min	P90/P10
Hospitalization rate due to asthma per 10,000 (all ages)	8.4	99.1	2.4	4.8
Hospitalization rate due to asthma per 10,000 (0–14)	15.6	349.3	0.0	10.1
Hospitalization rate due to asthma per 10,000 (15–64)	4.2	43.6	0.3	6.0
Hospitalization rate due to asthma per 10,000 (65 and over)	24.7	186.1	7.0	4.3

**Table 3:** Correlation matrix of dependent and independent variables.

Bivariate Association	Rate (All)	Rate (0–14)	Rate (14–64)	Rate (65+)	NHI Prem.	PCP	Beds (<300)	Beds (≥300)	CO	SO_2_
NHI prem.	−0.290 **	−0.150 *	−0.371 **	−0.363 **						
PCP	−0.220 **	−0.110	−0.245 **	−0.333 **	0.498 **					
Beds (<300)	0.428 **	0.316 **	0.453 **	0.334 **	−0.160 *	0.212 **				
Beds (≥300)	−0.055	−0.001	−0.064	−0.138 *	0.219 **	0.545 **	0.208 **			
CO	−0.191 **	−0.117	−0.223 **	−0.236 **	0.314 **	0.194 **	−0.077	0.037		
SO_2_	−0.005	0.058	−0.071	−0.086	0.391 **	0.311 **	0.050	0.179 **	0.436 *	
PM_10_	−0.039	−0.033	−0.040	−0.026	0.035	0.038	−0.115	−0.056	0.183 *	0.286 *

* *p* < 0.05, ** *p* < 0.01.

**Table 4:** Regression analysis of hospitalization rates for asthma by age group.

	All Ages		0–14		15–64		65 and Over	
	Coefficient	SE	Coefficient	SE	Coefficient	SE	Coefficient	SE
Baseline (intercept)	14.613	4.609	26.996	19.653	9.226	2.403	46.322	10.030
Average NHI premium per household	−0.050	0.040	−0.060	0.171	−0.050 *	0.021	−0.156	0.087
Primary care physicians per 10,000 population	−1.901 ***	0.468	−5.038 *	1.995	−0.959 ***	0.244	−4.833 ***	1.018
Hospital beds per 1000 population (<300)	1.693 ***	0.216	4.721 ***	0.921	0.945 ***	0.113	3.036 ***	0.470
Hospital beds per 1000 population (>300)	0.032	0.189	0.265	0.808	0.022	0.099	−0.068	0.412
Annual average ambient concentration of CO	−11.566 *	4.974	−35.749	21.211	−5.868 *	2.594	−27.768 *	10.825
Annual average ambient concentration of SO_2_ (X100)	8.961 *	3.752	36.375 *	15.999	3.155	1.956	14.193	8.166
Annual average ambient concentration of PM_10_	0.013	0.076	−0.059	0.326	0.019	0.040	0.090	0.166
Adjusted R^2^	0.293		0.155		0.343		0.295	

* *p* < 0.05, *** *p* < 0.001.

## Appendix A

**Table A1:** Characteristics of independent variables.

Variables	Mean	Min	Max	Std. Deviation
Average NHI premium per household	73.7	47,1	144.2	16.0
Primary care physicians per 10,000 population	3.1	0.0	12.7	1.5
Hospital beds per 1000 population (<300)	3.5	0.0	14.4	2.5
Hospital beds per 1000 population (>300)	1.6	0.0	26.6	3.2
Annual average ambient concentration of CO	0.48	0.26	0.93	0.11
Annual average ambient concentration of SO_2_ (X100)	0.44	0.15	1.04	0.16
Annual average ambient concentration of PM_10_	47.8	30	71	6.9
